# *‘The Words Will Pass with the Blowing Wind’:* Staff and Parent Views of the Deferred Consent Process, with Prior Assent, Used in an Emergency Fluids Trial in Two African Hospitals

**DOI:** 10.1371/journal.pone.0054894

**Published:** 2013-02-11

**Authors:** Sassy Molyneux, Maureen Njue, Mwanamvua Boga, Lilian Akello, Peter Olupot-Olupot, Charles Engoru, Sarah Kiguli, Kathryn Maitland

**Affiliations:** 1 Health Systems and Social Science Research Group, Kenya Medical Research Institute -Wellcome Trust Research Programme, Kilifi, Kenya; 2 Centre for Tropical Medicine, University of Oxford, Oxford, United Kingdom; 3 The Ethox Centre, Department of Public Health and Primary Health Care, Oxford University, Oxford, United Kingdom; 4 Kilifi Clinical Trials Facility, Kenya Medical Research Institute-Wellcome Trust Research Programme, Kilifi, Kenya; 5 Wellcome Trust Centre for Clinical Tropical Medicine, Department of Paediatrics, Faculty of Medicine, Imperial College, London, United Kingdom; 6 Department of Paediatrics, Mulago National Referral Hospital, Makerere University, Kampala, Uganda; 7 Mbale Regional Referral Hospital, Mbale, Uganda; 8 Soroti Regional Referral Hospital, Soroti, Uganda; 9 Malaria Consortium, Kampala, Uganda; Nottingham University, United Kingdom

## Abstract

**Objective:**

To document and explore the views and experiences of key stakeholders regarding the consent procedures of an emergency research clinical trial examining immediate fluid resuscitation strategies, and to discuss the implications for similar trials in future.

**Methods:**

A social science sub-study of the FEAST (Fluid Expansion As Supportive Therapy) trial. Interviews were held with trial team members (n = 30), health workers (n = 15) and parents (n = 51) from two purposively selected hospitals in Soroti, Uganda, and Kilifi, Kenya.

**Findings:**

Overall, deferred consent with prior assent was seen by staff and parents as having the potential to protect the interests of both patients and researchers, and to avoid delays in starting treatment. An important challenge is that the validity of verbal assent is undermined when inadequate initial information is poorly understood. This concern needs to be balanced against the possibility that full prior consent on admission potentially causes harm through introducing delays. Full prior consent also potentially imposes worries on parents that clinicians are uncertain about how to proceed and that clinicians want to absolve themselves of any responsibility for the child’s outcome (some parents’ interpretation of the need for signed consent). Voluntariness is clearly compromised for both verbal assent and full prior consent in a context of such vulnerability and stress. Further challenges in obtaining verbal assent were: what to do in the absence of the household decision-maker (often the father); and how medical staff handle parents not giving a clear agreement or refusal.

**Conclusion:**

While the challenges identified are faced in all research in low-income settings, they are magnified for emergency trials by the urgency of decision making and treatment needs. Consent options will need to be tailored to particular studies and settings, and might best be informed by consultation with staff members and community representatives using a deliberative approach.

## Introduction

### Alternatives to Full Prior Consent in Emergency Research

Emergency research involves people with a life-threatening medical condition that requires urgent intervention, and emergency trials are important to allow relevant interventions to be evaluated. Given that most interventions used in the treatment and management of critically ill children have at best only been tested in animals or in adults, there is a need for emergency paediatric trials to advance evidence-based paediatric practice, and child health and well-being [1].

In emergency paediatric research, prior consent by parents or other legal guardians is recommended by the EU, but is recognised as challenging given the urgency of the patient’s condition, and the difficulty for the guardian of assimilating trial information in such distressing circumstances [1], [2]. Current regulations in some European countries and in the United States allow for alternatives to full prior individual consent in emergency research, while guidelines elsewhere in the world vary or are not specifically addressed [3]. The FDA allows for alternatives to full prior individual consent only in specific situations, as follows [4]:

The subject has a life threatening condition that necessitates urgent intervention;
Available treatments are unproven or satisfactory;
Consent from the subject (or a surrogate) is not feasible due to the urgency of the patient/subject’s condition;
The research could not otherwise be performed;
The risks and benefits are reasonable;
There is a prospect that the trial will be of direct medical benefit to the patient/subject; and
There is consultation with the community from which subjects will be drawn, including public disclosure of the study design and risks prior to commencement.

These conditions have been argued to be unnecessarily restrictive, and to have contributed to a decline in the amount of emergency research.

A range of alternatives to full prior individual consent have been suggested, with deferred consent distinguished here from retrospective assent through including prior assent. Assent refers to an affirmative agreement; an agreement which implies at least a minimal level of understanding and ability to indicate a choice (Table 1; drawing on [1], [5], [6], [7]). Each alternative option has its limitations. While there is no consensus on the most appropriate approach, it is generally agreed that full information must be given to surrogates as soon as possible, and that guidelines and regulations that apply to all clinical studies must be adhered to [8], [9], including having a Data Safety and Monitoring Board. Ideally, emergency research should also be reviewed by committees with special training [1], [10].

**Table 1:** Alternatives to prior individual consent in emergency paediatric research.

Type of consent	Characteristics	Critique
**Deferred consent**	Initial assent to enter the study is obtainedFull informed consent is deferred (delayed) until the patienthas stabilised and/or surrogates areable to listen and understand trial informationSurrogates can withdraw at any point	Consent cannot be obtained after the intervention has already been given; consent is only therefore permission to remain in the study
**Proxy consent by** **third parties**	An appropriate person other than the parent/immediate guardiangives consent for the participantPotential proxies include - legal representatives, and independent physicians	Difficulties in ensuring independence of proxiesProxies not capable of knowing the wishes of a parent especially without prior discussion
**Advanced consent/Presumed consent:**	Potential participants are identified prior to meeting eligibilitycriteria and consent for future enrolment should theybecame eligible	Difficult to identify participants in advance in emergency researchPotentially causes unnecessary distress or harm, especially where inclusion criteria are not met
**Retrospective** **consent**	Initial research intervention occurs without the surrogate’s consentConsenting takes place after the initiation or completion ofthe research intervention	Participants have no control over what has been done in the past
**Waiver of consent:**	Research intervention occurs without the participant’s orsurrogate’s consent	Considered to be a violation of patient autonomy

Given the challenges in obtaining full prior consent in paediatric emergency research, and variability and controversy in current guidelines and regulations [3], documentation and exploration of alternative approaches to obtaining informed consent in emergency research involving children is needed. We therefore conducted a social science sub-study alongside an emergency paediatric care trial to document and explore the views and experiences of key stakeholders – trial team members, health workers and parents - regarding the consent procedures.

### The Consent Process in the FEAST Trial

The FEAST (Fluid Expansion As Supportive Therapy) trial was an emergency paediatric trial aimed at identifying the best strategies for treating and managing critically sick children on admission to hospital with severe febrile illness and shock. The trial was conducted in six hospitals in three East African countries (Uganda, Tanzania and Kenya), and involved 3170 critically ill children – with an estimated mortality rate of 15% - aged between 2 months and 12 years. The trial was a 3-arm randomised open comparative trial of fluid resuscitation strategies with children randomised into: (1) Immediate volume resuscitation with normal (0.9%) saline; (2) immediate volume expansion with 5% human albumin solution (HAS); or (3) control: no immediate volume expansion. The trial has been described in detail elsewhere [11].

The trial team and colleagues drew upon existing guidance and commentaries to develop a consent process which included written prior consent where possible, but an option for deferred consent with prior assent for a sub-set of children who needed immediate resuscitation (where prior consent was not possible), or where guardians or parents were not available or were perceived to be so distressed they were unable to receive or understand information (Table 2).

**Table 2:** Criteria for deferred consent in FEAST Study [3].

Degree of Emergency	Consent Status
**Pre-terminal**	Deferred consent
**Immediate resuscitation:** other life threatening complications e.g. seizures, hypoglycaemia, hypoxia	Deferred consent
**No other life threatening complications:** parents able to receive and understand information	Full informed consent prior to enrolment
**No other life threatening complications:** Guardian or parent not available or; parent or guardian unableto receive or understand information	Deferred consent

In the FEAST trial, therefore, deferred consent involved a verbal assent from parents or guardians prior to enrolment (see Table 3 for information included) and a delayed full informed written consent after the child had stabilised and when it was perceived that parents were better able to receive, evaluate and discuss the information. The intention was that verbal assent allowed parents to make a decision about trial participation on the basis of brief information, and gave them an opportunity to ‘opt out’. For the children who died shortly after verbal assent on admission, and before full consent, parents were not approached for retrospective informed consent. As elsewhere [2], [12], [13], obtaining consent from bereaved parents was considered to potentially further distress parents at a profoundly emotional time, possibly resulting in parents blaming themselves for permitting research participation [3].

**Table 3:** Phrasing of assent in the deferred consent process [3].

We are going to provide the treatment for your child that is recommended by the government.
We want to find out if we can improve on these current recommendations by trying new treatments that we think will work better. We do this by research
All research is checked by independent committees to make sure that the potential benefits to individuals outweigh the risks. All participation in research is voluntary, and so you can refuse
We would like your child to participate in research for us to learn the best way to give fluids to very sick children.
Do you agree for your child to take part in this research? You can say no and your child will still receive the same level of care with the governments recommended treatment.

An intensive training and retraining process was conducted for the FEAST trial by trained facilitators, including specific modules on assent and consent. Interactive training methods drew upon trainees’ knowledge and experience to contribute ideas for handling potential field scenarios, within an institutionally agreed consent SOP [14]. The time required for verbal assent was generally 2–5 minutes and could be obtained at the bedside, even whilst other aspects of emergency care were being administered (e.g. airway, oxygen, hypoglycemia correction). Consent required the full attention of the parent or guardian and generally took 20–30 minutes depending upon the amount of discussion. The latest international guidelines [15] recommend rapid and prompt fluid resuscitation of shock within 15 minutes of diagnosis, and the WHO guidelines recommend treatment ‘as quickly as possible’ [16], illustrating the importance of the deferred consent option for some parents (Table 2). Consent processes were reviewed regularly by monitors, and staff were encouraged to raise any challenges in day to day trial administration to their line managers or to the trainers throughout the trial. As part of community engagement, trial specific information was shared with key communities before the trial began, with key communities defined as ward parents, and hospital ward staff and managers. Widespread information giving in communities was opted against given the large size of hospital catchment areas, the small proportion of children who would ultimately be eligible for the trial, and the potential for complex information given in large community meetings to cause unnecessary concerns about attending hospitals for care.

In this paper we present the findings of the social science sub-study of the FEAST trial, and consider the implications for similar trials in such settings in future. We do not seek to evaluate or discuss the ethics of the trial, which was approved by science and ethics committees in four countries (UK, Uganda, Kenya, and Tanzania).

## Methods

### Study Sites – two FEAST Trial Hospitals

The consent study was conducted in two FEAST trial sites: Kilifi District Hospital (KDH), Kenya and Soroti Regional Referral Hospital (SRRH), Uganda. The sites were purposively selected to offer differing experiences based on different background situations, levels and types of trial inputs into the site (Table 4), and therefore perceived individual benefits.

**Table 4:** Key features of the two FEAST trial sites.

Site	Kilifi District Hospital (KDH), Kenya	Soroti Regional Referral Hospital (SRRH), Uganda
Size of paediatric wards	42 beds	62 beds
Experience conducting trials	Clinical trials conducted for over 20 years	First major research activity
Community engagement activities	Coordinated community engagement activities focusing onthe institution and (where appropriate) specific studies	No formalised community engagement strategies for research
Employment and trainingof staff	Most staff within pre-existing clinical research group	Most staff involved in the trial trained and recruited specifically
Refusal rates for trial	High relative to other sites	Low relative particularly to KDH
Hospital user charges andother costs (a)	National exemption of charges for under five year olds, but little policy adherence.Food is provided for patients and most basic consumables available at a cost.	National exemption of charges for all admissions, but little policy adherence.Relatives provide and cook food in hospital grounds, and purchase many consumables needed (e.g. gloves, intravenous lines, some medicines) from local shops.
Trial inputs into study site	Established clinical programme as a robust platform forclinical trials (b). Also, trial provided extra personnel foremergency triage and patient monitoring, additionaldiagnostic tests and service-wide training inemergency care.	Basic maintenance and painting of paediatric wards, emergencyand triage equipment and training for all staff, and employmentof additional personnel.
Trial benefits forindividuals (d)	Close observation, treatment of new illnesses identified during admission, and free treatment of minor illnesses post discharge up until the 28-day follow-up visit.	As with KDH, every trial participant was allocated a dedicated‘clinical trial pack’(c).

(a) See [32], [33] for information on lack of adherence to user fee policies.

(b) Includes substantial support to the hospital for medical personnel (doctors, clinical officers, nurses and ward assistants); paediatric drugs, devices and equipment. Research funds also support the construction and running of an 8-bed paediatric high dependency ward available to all paediatric admissions, regardless of research involvement.

(c) Including cannulae, syringes, infusion sets, antibiotics, anti-malarials and blood testing consumables. Not needed in KDH where such support is provided to all inpatients in HDU.

(d) The usual hospital admission fees for the participants were not waived during the trial, in an effort to ensure that parents did not feel obliged to join the trial to save money. However, in Soroti only a few patients in a semi-private room are charged fees by the hospital.

### Consent Sub Study Data Collection and Analysis

We held interviews with parents and trial staff and health workers between June and December 2010, before the trial was stopped early in January 2011 by the Independent Data and Safety Monitoring committee because of lack of benefit and potential harm from bolus fluid in African hospitals compared to control [11].

#### Interviews with parents

34 in-depth interviews were held with parents of participating children (15 in Kilifi; 19 in Soroti); and five with parents who had refused (all in Kilifi). In addition, we interviewed 12 parents of children concurrently admitted in the wards but not involved with the trial in Soroti. The equivalent figure for Kilifi was 138; a higher figure because these interviews were being conducted as part of a broader study. Trial participants were identified from trial data, and other parents on discharge from the wards. Inclusion of non-study parents was aimed at identifying whether issues raised by trial participant parents were more about high dependency ward experiences than the trial itself. Parents of children who had died were not included due to the very sensitive nature of such interviews. Interviews with parents were conducted in parents’ preferred language, by trained staff.

#### Interviews with trial staff and health workers

We interviewed 30 staff involved with the FEAST trial (15 in Soroti and 9 in Kilifi) individually, and held two group discussions with health workers (primarily nurses) in Kilifi (N = 15). Six additional individual interviews were held with hospital staff and managers. All staff interviews were conducted in English by SM and MN.

All interviews were recorded, transcribed, and - where relevant - translated. Data were analysed using a modified framework approach [17]. Data were imported into NVIVO 9, and organised using a coding tree developed by SM and MN based on pre-study areas of interest and independent reviews of a sub-set of transcriptions. Charts by theme allowed comparison of issues identified by site and type of interviewee.

### Ethics Statement

The consent sub-study of the FEAST trial was approved by National Ethics Review committees in Kenya and Uganda (the Kenya Medical Research Institute Ethics Review Committee and the Makerere University Faculty of Medical Research and Ethics Committee respectively). Written consent was sought from all respondents. Interviews were conducted independently of the trial team, led by researchers with a long interest in consent processes in East Africa [18], [19], [20]. All recordings, transcriptions and quotes have been anonymised to protect confidentiality.

## Findings

Following an overview of the consent rates and types across the two sites, we present staff and then parent perceptions of the consent process, followed by two factors influencing perceptions: parental understanding and decision-making in the trial, and factors influencing this.

### Rates and Types of Consent in the Study Sites

Information on consent rates and type was collected as part of the trial procedures (Table 5).

**Table 5:** FEAST trial consent rates and types of consent.

Type of consent	Overall	Kilifi	Soroti
**Total Enrolled**	**3170**	**216**	**633**
Assent Only (% of enrolled)	135 (4%)	4 (2%)	36 (6%)
Full prior consent (% of enrolled)	1534 (49%)	189 (87%)	257 (40%)
Deferred consent following assent (% of enrolled)	1501 (47%)	23 (11%)	340 (54%)
**Total eligible** *	**3463**	**339**	**660**
Refused consent (% of eligible)	293 (8.5%)	123 (36%)	27 (4%)

* Not including those that met inclusion criteria but were not enrolled for other reasons such as trial packs not being available. Refer to FEAST Trial paper for more information on ‘other’ reasons for non-enrolment.

Across all six trial sites, 49% of parents gave full prior consent and 47% assent prior to randomisation as part of a deferred consent process. A small proportion (4%) only gave assent, with these patients dying before deferred consent could be sought. Only a tiny fraction (0.4%) of parents who initially assented or consented to participation in the trial later withdrew their children.

The number of children enrolled in the trial was higher in Soroti (n = 633; contributing 20% of total trial participants) than Kilifi (n = 216; contributing 6.8%). We found substantial and statistically significant differences between Kilifi and Soroti in rates of consent and in the proportion with prior assent. 87% of participants’ parents in Kilifi gave prior full consent (189/216); whereas in Soroti the equivalent proportion was 40% (257/633) (chi-squared p<0.0001). Just over half of parents in Soroti (54%) and 11% in Kilifi (p<0.0001) went through the prior assent with deferred consent. In Kilifi, a far higher proportion of screened and eligible participants refused consent (36%; 123/339) compared to Soroti (4%; 27/660) (p<0.0001).

### Staff Perceptions of the Consent Process

#### Positive views

The approach to consent developed by the trial team was generally strongly supported by all staff, with some nurses in Kilifi describing the consenting process as fostering rapport, trust and communication with patients. It was felt to be important to have prior full consent wherever possible to avoid ‘*depriving the mother of the full information that she needs to make an informed decision’.* Including assent for the critically ill, as opposed to a waiver or retrospective consent, was felt to be important because the research involved an intervention (fluid boluses) that was not standard of care, and therefore some information and an ability to opt out for parents was required:

*I think we should [assent] because this is not standard treatment that you are giving; you are doing research and you don’t know for sure whether what you are giving is good or whether it’s bad…(Soroti\Staff\ST011).*

Most staff involved in administering a deferred consent process with verbal assent were administering this form of consent for the first time and viewed it very positively. They appreciated that it allowed emergency treatments to be started without delay:

*I felt that the hastened bit of it [referring to assent] was quite appropriate… because when the patient is that sick you also feel for the patient and you feel for that mother. [You think] ‘What if something happens before I start these fluids while maybe I am talking to this mother?’ (Kilifi\Staff\KL011).*

Also regularly mentioned by staff was that including an option for verbal assent allowed them to continue with the research in a way that protected them from any later blame from parents for having enrolled children:

*There are people who are really difficult. When you start doing something on the child and the child dies, then there you will be stuck: somebody can start saying that had it not been because of these [study procedures], my child would not have died…. (Soroti\Staff\ST012).*

*We have to tell them whatever is happening because if you don’t tell them and something happens, the blame will be on us (Kilifi\Staff\KL005).*

Deferred consent (following initial verbal assent) was also described as ensuring that another formal step to support parents’ understanding and their ability to withdraw was introduced:

*You know even you if you are in a state of shock (laughing) you may say yes to something if you are not mentally stable… you may say yes then later you say no when you came to that part of filling in the consent form… at that point it will be wise for you to withdraw that child from the study and it’s really understandable… it is very important to go back as soon as possible (Soroti\Staff\ST009).*

A final argument often raised in favour of deferred consent was that it can allow some ‘buying of time’ to wait for key decision makers to arrive when children are brought to hospital by relatives other than their parents, or by a mother who does not want to make a definitive decision on her husbands’ behalf:

*…depending on families there are mothers who can make decisions by themselves so those ones can accept, but there are these ones who rely on the father as the one to make decisions - majority of them go for the assent (Soroti\Staff\ST012).*

This latter point was challenged by some interviewees, particularly in Kilifi, where there was some lack of clarity on whether or not there was a study-specific ‘arrangement’ to allow research to proceed in these circumstances. Some Kilifi-based interviewees were concerned that this would not be permitted in ethical practice more globally, and about how the child’s parents might later react if they heard their child had been assented by somebody else:

*When it comes to relatives assenting or consenting for patients who are not their children it’s a bit tricky and because this child is not theirs… you never know how the parents will react, so I think an assent cannot…[be] for somebody who is not present (Kilifi\Staff\KL011).*

#### Challenges and concerns shared by staff

The main challenge raised by staff was the validity of either assent or full consent when given by guardians during admission of a critically sick child:

*Remember you are getting somebody in a very desperate situation and you are saying, okay I have screened this patient, and this patient is eligible now we have such and such a trial but at this stage I just need you to tell me whether you are willing or not willing to participate. Now to somebody who is desperate I think a yes answer is bigger than a no answer…the desperation here is not … to enter the trial but somebody is desperate to save the life of a very critically sick person (Soroti\Staff\MB001).*

The above quote hints at parents feeling like they have to agree to ensure their children receive all emergency treatment rather than the study intervention. Other challenges highlighted by staff were parents not being able to listen given their anxiety, and – overlapping with both perceived pressure to agree and with difficulties in concentrating and understanding the information – parents believing that by agreeing to trial enrolment that all children would receive a fluid bolus (as opposed to no-bolus, the control). The bolus was reportedly seen as primarily to benefit their child.

A potential risk regarding deferred consent raised more often in Soroti than in Kilifi was that the option could be overused; introducing ‘a laziness’ in staff. In Kilifi, some staff argued that assent could ultimately lead to more late refusals, with parents having less interest in continuing to be in the study and withdrawing permission once their child had begun to improve (the point at which full consent is sought). This was difficult to explore quantitatively, given the small numbers with deferred consent in Kilifi.

In both settings staff reported that even the assent process introduces delays through encouraging questions, which introduces a dilemma in delaying initiation of treatment. Apparently unclear among staff was exactly what information they should give during assent. Although SOPs include the information, and this was covered during training, some staff reported that what to say was ‘*really much more left to the prerogative of the clinician or the nurse’.* Some staff were also unclear about whether or not an actual ‘yes’ was required; reportedly a major challenge in both settings where mothers often told staff to ‘*just do what you want*’:

*These mothers come with all their anxiety, the child is sick. As much as someone would try to look brave and to say that I have actually understood go ahead - … mostly they say ‘do what you can, what I want to see is my child well’ (Kilifi\Staff\KL011).*

The challenge was whether to accept such comments as a form of implicit agreement. Also unclear to staff was how to handle mothers who want to wait for a father to decide on study participation. In both settings fathers are often the main decision-makers in households but may not be present when the child is admitted. The dilemma is whether or not the mother is using the husband as a way to politely refuse; as a form of ‘silent refusal’:

*It’s quite hard because often the reason you are given is that maybe ‘huyu ni mtoto wa wenyewe’ [this is the owner’s - i.e. the father’s - child]…., ‘I need the father also to give consent’ or that ‘I am waiting for the father’. So often they will not really come out and tell you that it is KEMRI [that is worrying me] but they always give other reasons (Kilifi\Staff\KL008).*

One Kilifi staff member described asking the mother if she herself felt comfortable with the trial, and if so, suggesting that the child was enrolled and that the husband be fully informed on arrival. Because when fathers do later come, they *‘are usually not problematic so it’s just the fear the mothers have but the fathers are usually quite positive, quite ok to accept’.* However some parents’ comments illustrate the potentially serious consequences of going against a father’s wishes:

*you don’t know how they are living at their home………that [ie a woman deciding in the absence of a husband] may cause her to be slapped (laughs)… because the husband may want to know why she has done that; why she didn’t wait for her husband to come…. That may cause chaos (Kilifi/Parent/KLF 004).*

The provision in the FEAST trial to waiver full consent if a child died following enrolment by parental assent, was understood and supported by most staff. They commented that it would ‘not be good’ or be ‘unethical’ to go back, given the sensitivity of the situation. Parents are typically crying, and sometimes collapsing. However, one staff member thought it was a study requirement to retrospectively obtain full consent even in the event of a death. Several felt that although it would be *‘harsh because someone has just lost their child and they are emotionally upset and all that’;* it would have been better to obtain consent retrospectively to protect staff legally.

### Parents’ Recall and Perceptions of the Informed Consent Process

As might be expected from staff comments and the medical condition of many children, parents’ descriptions of the admission process and information given at that stage focused on their concern for the child’s health and for their desire for treatment to proceed as fast as possible. Many parents said they ‘cannot remember’ or ‘have forgotten’ what they were told, or that they were ‘not listening to anything’. As one mother described in Soroti:

*You know, you can also forget because the things [they told me] were too many and you know being only one head my heart was shaken about how the child was…that’s why [my head] didn’t grab many things (Soroti/FEAST mother/ST 010).*

Others had some recollection, sometimes quite detailed, of the consent process and of the content of the discussion, but even these relatively detailed descriptions illustrate some lack of understanding or recall of the relevant trial details, and a hope or belief that inclusion in the trial is positive for the child. Two examples:

*It’s a person’s freewill. If you agree to join that group, something like that, that’s when your child will be put some water but if you refuse, your child will not. That’s why she asked me before they put the water in the child and asked me whether I agree or not but I considered my child’s condition and said it’s alright.* ***But the water is really helpful to the child.*** *(Kilifi/Parent/KLF 011; authors’ emphasis).*

*They said that they are working with Makerere University and they have come to bring a study that involves treating people using fluids or something like that. They told me that it’s not only here, it is also being done in other places and countries including even some countries in Africa and others outside Africa. They are trying to treat children who are very ill using fluids. They also said that the way they have seen in some countries, it seems it is good so they also want to try it in Uganda to check if it is good so that they can see whether to continue that kind of treatment. Then they asked me that do you accept to be with us in this study? That they would treat the child and at the same time studying if I agreed. So I said that I agree, then they started treating the child from that day up to today,* ***the child is still in their hands*** *(Soroti/FEAST Father/ST 011; authors’ emphasis).*

Many parents appreciated having been given some information before treatment began, but some felt – sometimes quite strongly - that it was pointless or even an additional worry to be given information and especially being asked ‘opinions’ (i.e. consent) at the stage:

*Understanding, [is not something that can happen] until somebody sees her child is fine or recovered, but if you came to me and announce or talk, shouting to me explaining to me about KEMRI when my child is ailing...* ***I won’t listen to you, it’s like you will be talking to the wind and the words will pass with the blowing wind*** *. Because to me I will be looking on the condition of my child; not listening to you (Kilifi/Parent/KLF 016; authors’ emphasis).*

*What was not appropriate or what didn’t please me was that of being consulted first … I felt bad because you have come all the way from home because your child is not feeling well, you have brought the child and* ***instead of them taking an urgent step of treating the child they start asking for your advice*** *…. If it were not my decision I wouldn’t have brought the child to this place… (Kilifi/Parent/KLF007; author’s emphasis).*

Another mother’s comment suggested that the way that consent was administered may not always have been ideal:

*I was seated but my child was on a bed somewhere aside… she was even explaining while I had stood up to look at my child. In the end she [the nurse] asked them [the other nurses] to block me from seeing my child [so I would listen]. I cannot complain because they were in a hurry so as for the child to be put [on] the [infusion of] water (Kilifi/Parent/KLF 011).*

In Kilifi, some of those who refused suggested that the consent signature indicated a handing over of the child; an absolution of blame on all sides ‘*like if anything happens I don’t want us to blame each other’* which was felt to be deeply uncomfortable given the severity of the child’s illness:

*You are told it is your decision. That is what is making people refuse because instead of just treating the child so that he/she is cured … It’s like you are saying in case there is anything that happens, I don’t want us to blame each other. Because you will have signed and you won’t have anything to say because you have signed yourself. So that is why people are refusing (Kilifi/Parent/KLF007).*

The overwhelming recommendation from parents was for the treatment to come first and the talking and explaining later:

*When a child arrives there, let him/her be treated first and then later let [the parents] be asked the questions. I even told the nurses that but they said if you have not yet decided ‘yes’ or ‘no’, we can’t start giving the child water. Now, because I want the child to recover I have to say ‘yes’ so that they can start the water (Kilifi/Parent/KLF 013).*

### Factors Influencing Parental Decision-making

Many of the above comments, including from staff, illustrate the difficulty of differentiating a trial related treatment intervention from standard of care. Looking across all parents’ responses to questions during our interviews, only 7/39 (18%) appeared to recall the nature of the research they were involved in, 23/39 (59%) recollected some elements of the research, and 9 (23%) simply described their whole experience as clinical care (Table 6). Those with good or some recall described a ‘project’, voluntary participation, and non-routine ‘water’ treatment. For some, simply being asked (i.e. consent) triggered their knowledge that they were in a research project.

**Table 6:** Parents’ recall on discharge that their children were in a study.

Levels of Understanding	Kilifi (n = 19)	Soroti (n = 20)	Total (n = 39)
Clear understanding	3	4	7
Some elements of trial understood	13	10	23
No apparent distinction from clinical care	3	6	9

Clearly difficult for parents and staff was explaining and understanding the concept of a trial and ‘equipoise’, leading to many parents suggesting that a decision, and particularly saying ‘no’, did not make any sense:

*If you refuse what will you do in that condition* ***and I have followed you*** *,* ***you cannot refuse*** *. If I were to refuse, I couldn’t have come with him to the hospital (pause) we are grateful. (Kilifi/Parent/KLF 011; authors’ emphasis).*

The above perception may have been contributed to by many of the trial staff apparently not being in equipoise at the time of this consent sub-study, with many believing that the intervention arms would prove to be life saving. As one staff member reported:

*we have come to appreciate that at least that rapid infusion of fluids within the first one hour for very sick children really has some benefits so we are even trying to do it informally not only for the FEAST children but for some other children… it has helped us at least come to that conclusion even before the study has concluded (Soroti\Staff\ST009).*

Others were concerned that refusal might lead to poorer quality of care, with one parent in Soroti mentioning you would have to ‘*go to the other side where you have to buy things*’. This might relate to the FEAST patients being relatively distinguishable from others in the ward not participating in the trial in Soroti due to their physical location and also because enrolled children did not have to buy prescribed medicines or infusion lines as often happens for paediatric patients (see Table 4). This difference contributed to trial participants being perceived by parents as getting much better quality of care.

*Once you are in that room [study room] you are given everything even the cannula, quinine, the drugs…All you do is to just look and appreciate that the child is being treated. The only expenditure is on what you have to eat and for the child because you see that even life is getting better … Even the nurses are good and the help offered is okay (Soroti/Non-FEAST mother/ST 005).*

*you can wish in your heart that if only you were also the one being treated like that, maybe it would be better (Soroti/Non-FEAST Mother/ST 001).*

Certainly, in both settings, participants’ parents greatly appreciated the close monitoring and quick attention given to their children while at the ward, and regularly discussed being impressed and pleased by the concern and ‘tender care’ shown to children by the nurses and doctors.

Parents’ relatively low exposure to science and research was often mentioned by staff as contributing to parents’ inability to differentiate between research and standard care. In Kilifi, previous information about or personal experience with research studies in the wards or in the community sometimes led to parents automatically refusing the trial, either because they were aware that research participation is voluntary and had no interest in participating, or through a general concern about the institution’s research. The latter was sometimes based on rumours that have been extensively described in Kilifi and elsewhere [18], [20], including misperceptions that blood samples are mixed and sold, or that staff are ‘devil-worshippers’. In other cases, past experience or information about KEMRI was much more positive, and appeared to lead to an automatic yes, regardless of understanding of study details:

*I told him that I know KEMRI; one of my grandchildren is in a KEMRI malaria vaccine study so I know this organisation. When he asked me whether I was going to join or not I told him that I’m happy to and I joined (Kilifi/Parent/KLF017).*

## Discussion

There is widespread agreement on the need for emergency paediatric research, arguably particularly in low-income settings such as sub-Saharan Africa where a disproportionate burden of child mortality occurs within hospitals, often within hours of admission. An important challenge with regards to such trials is developing an appropriate consent process. The interviews we held with those most closely involved with the FEAST trial reveal the significant challenges faced in meeting all of the key elements of consent: parents are seriously anxious and vulnerable; and staff are stressed by balancing the urgent need to proceed with treatment with a concern about being later blamed by parents for encouraging them to join a trial without meaningful initial consultation. This latter point is important given staff recognition of the potentially pivotal role they play in handling a high-risk group of children.

### Differences in Consent Types by Site, and Views of the FEAST Consent Approach

Contextual differences between Kilifi and Soroti potentially contributed to less deferred consent and more refusals in Kilifi (Table 5). Contextual differences include those laid out in Table 4, as well as children being perceived by staff as generally less severely ill in Kilifi (and therefore less likely to be eligible for deferred consent), other studies actively recruiting children at the time when FEAST was being conducted in Kilifi (impacting on both staff pressure and parent interest), staff being more familiar with and therefore comfortable with full prior consent in Kilifi, and possibly generally more male decision-makers being present in Soroti.

Overall, the consent approach adopted by the trial, with an option for verbal assent with deferred full consent was seen in both settings as potentially protecting the interests of both patients and researchers where full prior consent was difficult or impossible. This option was also appreciated for avoiding delays in starting treatment. Other benefits were described as the formal introduction of a two stage process supporting better understanding, allowing another opportunity for parents to withdraw, and allowing other relatives to be involved in the consent process. Important to note however is that these latter benefits could potentially also be incorporated into a full prior consent process.

Within a generally positive view of the consent process, particularly among staff members, there were also some serious concerns raised. A major issue - revealed through discussions primarily with parents - was that being asked to make choices and listen to information on admission potentially causes harm, through raising concerns and doubts at a time when parents are unable to listen, ask, understand, or challenge those they are seeking help from. Relatedly, is whether voluntary decisions can ever really be made in this emergency context.

### Alternatives to the FEAST Trial Approach to Consent and Areas Needing Further Deliberation and Resolution

The main suggestion or recommendation from parents was that researchers should ‘*just get on with treating their child*’, and worry about consent later. This suggestion from parents in our settings echoes views of parents in UK gathered through a postal questionnaire survey aimed at informing a proposed double blind RCT [13]. However, some aspects of our data suggest caution in *retrospective consent for all (* Table 1):

- From a moral perspective, parents have a right to make a choice on their own terms [1], because the expert does not know everything and cannot know what is best for each person in a medical trial. Some parents did refuse, particularly in Kilifi, suggesting that prior assent or consent does give some people a choice, and interviews with refusals did not suggest that parents regretted this refusal;
- Some parents appreciated being given some information and an opportunity to opt out, despite recognising the difficulties in understanding;
- Most staff felt that it would be inappropriate *not* to give parents any choice prior to starting the study, for both moral and – possibly more strongly - legal reasons;
- Parents who recommended mandatory retrospective consent did not always appear to have a full understanding that their child had been involved in a trial. Parents’ recommendations may have differed significantly if they had understood their children had been involved in a trial, and the implications of this, more fully.
- Over time, there is a possibility that in a context of some concerns about, and distrust in research, an awareness that children could be enrolled without any prior information or option to opt out, could undermine public trust in health services and in researchers.
- We did not interview a key group of parents; those whose children had died, whose views - if based on an understanding of the trial and its implications – may have differed significantly.

An alternative to retrospective consent would be to implement *deferred consent with a prior assent for all*. Parents’ descriptions suggest that regardless of the severity of the illness in clinical terms, a child entering into an HDU with an acute illness leads to such anxiety that all parents effectively meet the criteria for deferred consent with prior assent. If the assent process information included a clear option for full consent at the point of admission if parents wanted it, the parent would then have greater control themselves on the amount of information they received on admission, rather than leaving it to staff to assess suitability based on a predefined criteria, or staff making the subjective decision about levels of ‘parental distress’.

A challenge with the latter is staff members’ preference particularly in Kilifi for a one-step full consent process in advance in order to comply with all the legal requirements of research inclusion in one go. However full consent at enrolment is unlikely to meet the moral commitment to individual choice even if fulfilling the legal commitment [21]. This preference may also have in part been related to some lack of clarity among staff that the national ethics committee which approved the trial has been accredited by the Pharmacy and Poison’s Board (PPB) and the National Council for Science & Technology (NCST); and that the PPB and NCST have in turn been delegated the authority nationally to regulate clinical trials. The two-step prior assent followed by full later consent process for all, or possibly even a more continuous consent process [22], with an option for parents to choose full prior consent, potentially allows both the moral and legal approaches to consent to be met. An important problem is that the confusions, anxieties and delays introduced by parents being presented with and facing a choice on admission are not avoided.

Given the challenges with each option raised above, future similar studies would potentially benefit from more prior community consultation on the above options, and on further more specific issues raised by this study, as discussed below:

#### What to do in the absence of the father, or both parents

Consenting non-parents has been practised by others, where circumstances for deferred consent have included ‘physical absence of the patient’s relative [in such a circumstance]… as soon as relatives arrive, this circumstance is no longer valid, and hence consent should immediately be sought’ (Jansen, p 897). We found that ‘buying time’ does not necessarily build confidence in a moral commitment to the goals of consent processes, as opposed to a determination to achieve the study sample sizes.

Literature from this part of the world and our data suggest that there might be serious harms from proceeding with a trial in the absence of a parent or father’s approval, including domestic violence, mothers or grandparents being blamed for a child’s death, and households being split [18]. In situations with strongly inequitable power relations such as vulnerable clinical contexts [23], mothers also sometimes request to wait for their husbands as a way of ‘silently refusing’; as a way of exercising their own agency [24]. Although fears and concerns leading to silent refusals might be related more to a community or an individual’s lack of trust in research or in the research staff, rather than to issues specific to a trial, an ability to refuse is a right, even if it is based on apparently ‘irrational’ argument [1].

The challenge with accepting all deferrals of research decisions to fathers in settings like Soroti and especially Kilifi is that the majority of mothers bring their children to hospital without their husbands. It may also be that many fathers would regret their wives not having agreed to studies when they are later informed, particularly given that most trials have a positive impact on all participants – the trial effect – regardless of which arm they are in; a positive impact which was noted in the FEAST trial, and also [25] perceived by parents of FEAST trial participants, particularly in Soroti. Another challenge is the cost and size of studies and the potential to introduce bias, if all mothers wanting to wait for husbands is strongly adhered to. At this stage, without further information, it is difficult to justify *not* allowing mothers to wait for their husbands or for relatives to wait for parents; an approach which appeared to contribute to relatively high refusal rates in Kilifi. To argue otherwise would be to weigh cost against very real risks to mothers, and their ability to exercise choice; i.e. the moral goal of consent [26].

#### What does assent mean in the deferred consent process?

Beyond the issues raised above about who can assent, Kilifi staff in particular raised a concern about whether an affirmative ‘yes’ was needed from those providing the assent. With most parents overwhelmed and distressed by their child’s condition on arrival, ascertaining a positive response from some parents was difficult. Some staff felt that it was essential to have a clear go ahead while others felt that this should not be mandatory because it introduces additional unnecessary pressures and delays. One potential option moving forwards would be to consider the assent process as an opt out opportunity, where those who are generally opposed to any research can refuse, either openly or through the silent approaches noted above, regardless of their reasons. Here there would an acceptance of some loss of voluntariness in order to minimise the risks and distress associated with taking time to give a definitive response. A related challenge is the precise assent information that is given, and in particular how to distinguish between usual clinical care and interventions or procedures specific to clinical research in a way that is brief but clear. Clinical research, clinical care and quality improvement overlap so much that decisions are potentially rendered meaningless.

#### Should parents be consented retrospectively after the death of their child?

Current recommendations require that the relatives or guardians of a research subject entered into a clinical investigation with waived consent be informed of their participation even after death (21CFR50.24). The FEAST trial did not do this, for similar reasons to others. For example Jansen et al (2007) noted:

*‘Confronting relatives again with the event that their loved one died on the ICU can be seen as harm or burden… If we can say that confronting bereaved relatives represents additional burden, which we have the duty [as providers] to relieve or prevent, it seems morally correct to adopt policies that prevent seeking deferred consent from proxy’s after their relatives’ death’ (p897).*

Shilling and Young (2009) also showed that parents feared making a ‘wrong decision’ and living with the knowledge that the ‘wrong decision’ led to a poor outcome. In retrospect, this might have particularly applied to parents of children in the FEAST trial, given the unexpected negative findings, although as noted above there is a strong suggestion that there was a positive trial effect on all participating children. Our interviews with staff members suggest that choosing not to proceed with the full consent process after the death of a child was appropriate, humane and appreciated. However, we did not interview parents of children who had died, and there were some staff members who were concerned that parents should be informed primarily for legal protection reasons. Although staff could potentially be reassured about the legal issues, a challenge with not fully informing parents is the possibility in the longer term of parents perceiving that full information had been concealed from them, with potentially negative implications for trust and future research [13].

#### Do parents have a choice?

A particularly fundamental concern with meeting the moral commitment of consent is that of parents being so desperate or vulnerable that they feel ‘they have no choice’. Where this choice is based on an obvious disparity between the standard of care or costs for participants and non-participants, as was apparently more the case in Soroti, this raises potential concerns about undue inducement [27], and suggests the need for mechanisms to strengthen equity across participants and non-participants. This could be through for example increasing benefits that go to all ward patients as opposed to just participants; an approach aimed at fair benefits [28], [29]. However, this would in turn raise concerns about costs of such trials given the significant resource constraints in many hospitals, and the generalisability of findings to settings beyond the trial sites. Indirect benefits to all patients, such as on-site training for all hospital staff, professional development and general improvement of the organisation of emergency care are relatively realistic, but do not entirely remove inequities and this concern.

### Activities to Support Strengthened Consent Processes

Good training and consent support processes are clearly essential for staff involved in emergency trials, with an important set of communications skills required including being able to read and respond to the variability among parents (in terms of their mood, understanding and information needs at a given time), and being able to recognise silent agreements or refusals. The FEAST trial training and supportive supervision processes, including for consent, were greatly appreciated by staff, and are essential in all research settings given that frontline staff are constantly making ethically important decisions [14], [30]. Our data suggests that supportive supervision needs to be constant, and to be seeking out and responding continuously to challenges and areas lacking clarity as they arise. This support should continue after a trial is ended, possibly especially where there are unexpected findings: however carefully, equipoise has been explained in initial protocols and trainings, frontline staff may develop their own expectations and interpretations. Our data also suggest that consent training should include discussion and debate on ethical and moral approaches to consent, including: 1) national and international review processes, including why and how alternatives to full prior consent are approved, and how staff are legally protected; and 2) how best to ensure that on a day-to-day basis, the legal elements and requirements of a trial do not outweigh the moral [31].

### Conclusion

The overall approach of using a deferred consent process with a prior assent was generally supported, particularly by trial clinicians and nurses. Prior assent was seen as protecting the interests of both patients and researchers, including through minimising delays in starting treatment. The potential challenges were voluntariness being undermined through inadequate assent information being poorly understood, and any information giving on admission acting as a form of harm or disadvantage through causing real or perceived delays. Further challenges raised included what to do in the absence of a father, and what it means and what to do when mothers do not give a clear agreement or refusal. While these challenges are faced in all studies, they are magnified by the urgency of the situation, and the need to make rapid decisions.

Possible ways forward for consent for paediatric emergency research depend on the level of risk of a study, and on the context. For a minimal risk study a consent waiver or retrospective consent may be considered appropriate. For a non-minimal risk study like FEAST, one possible option is prior assent for all parents, with an option for full prior consent given as part of the assent information. This approach would consider assent essentially as an opt-out mechanism, whereby some loss of voluntariness is accepted in order to minimise the risks and distress associated with taking time to give a definitive response.

Regardless of the approach adopted, the importance of strong communication skills and support for all frontline staff, and their understanding of the moral bases for consent, and of the science and ethics review process and of the legal protections they have, is clear. Nevertheless, concrete decisions on ways forwards requires further discussion and reflection, including through consultation with staff members and community representatives, including men, using a deliberative approach.
